# Machine learning for early prediction of acute myocardial infarction or death in acute chest pain patients using electrocardiogram and blood tests at presentation

**DOI:** 10.1186/s12911-023-02119-1

**Published:** 2023-02-02

**Authors:** Pontus Olsson de Capretz, Anders Björkelund, Jonas Björk, Mattias Ohlsson, Arash Mokhtari, Axel Nyström, Ulf Ekelund

**Affiliations:** 1 Department of Internal and Emergency Medicine, Skåne University Hospital, Klinikgatan 15, 221 85 Lund, Sweden; 2 Department of Cardiology, Skåne University Hospital, Lund, Sweden; 3 Department of Clinical Sciences, Lund University, Lund, Sweden; 4 Division of Occupational and Environmental Medicine, Lund University, Lund, Sweden; 5 Department of Astronomy and Theoretical Physics, Lund University, Lund, Sweden; 6 Clinical Studies Sweden, Forum South, Skåne University Hospital, Lund, Sweden; 7 Center for Applied Intelligent Systems Research (CAISR), Halmstad University, Halmstad, Sweden

**Keywords:** Acute myocardial infarction, Emergency department, High-sensitivity troponin, Machine learning, Deep learning, Chest pain

## Abstract

**Aims:**

In the present study, we aimed to evaluate the performance of machine learning (ML) models for identification of acute myocardial infarction (AMI) or death within 30 days among emergency department (ED) chest pain patients.

**Methods and results:**

Using data from 9519 consecutive ED chest pain patients, we created ML models based on logistic regression or artificial neural networks. Model inputs included sex, age, ECG and the first blood tests at patient presentation: High sensitivity TnT (hs-cTnT), glucose, creatinine, and hemoglobin. For a safe rule-out, the models were adapted to achieve a sensitivity > 99% and a negative predictive value (NPV) > 99.5% for 30-day AMI/death. For rule-in, we set the models to achieve a specificity > 90% and a positive predictive value (PPV) of > 70%. The models were also compared with the 0 h arm of the European Society of Cardiology algorithm (ESC 0 h); An initial hs-cTnT < 5 ng/L for rule-out and ≥ 52 ng/L for rule-in. A convolutional neural network was the best model and identified 55% of the patients for rule-out and 5.3% for rule-in, while maintaining the required sensitivity, specificity, NPV and PPV levels. ESC 0 h failed to reach these performance levels.

**Discussion:**

An ML model based on age, sex, ECG and blood tests at ED arrival can identify six out of ten chest pain patients for safe early rule-out or rule-in with no need for serial blood tests. Future studies should attempt to improve these ML models further, e.g. by including additional input data.

**Supplementary Information:**

The online version contains supplementary material available at 10.1186/s12911-023-02119-1.

## Background

Acute myocardial infarction (AMI) is one of the major causes of death worldwide, and the most important consideration in patients presenting to the emergency department (ED) with chest pain. However, the vast majority of these patients do not have AMI, and for decades clinicians have tried to improve methods to rapidly identify or rule out AMI [1].

The ECG, blood tests of cardiac troponin, and patient history are the cornerstones of the ED evaluation of patients with possible AMI [2–4], and several rule-based algorithms have been created to improve diagnostic accuracy and speed. In this context, the 0/1 h European Society of Cardiology (ESC) protocol [5] has gained widespread acceptance. However, this algorithm has several weaknesses, including that only a minority of patients meet the criteria [6], that the same hs-cTnT cut-off is used in all patients, and that important factors affecting the hs-cTnT value are not accounted for, e.g. renal function, sex and age [7, 8]. Indeed, recent studies show that adding other blood biomarkers may improve the predictive value of hs-cTnT algorithms [7–10]. In addition, the ESC 0/1 h protocol require two hs-cTnT samples, taken one hour apart. A protocol allowing accurate management decisions already after the first blood test may decrease the length of ED stay for the patients and help reduce ED crowding. When applied alone, the 0 h arm of the ESC protocol identifies patients with an arrival hs-cTnT below 5 ng/L for safe and early rule-out, but these patients are relatively few [5].

Machine learning (ML) for the detection of acute disease is not new [11], but the introduction of deep learning has allowed these algorithms to emerge as powerful tools to predict complex phenomena with very high accuracy. These models could improve diagnostic performance compared to more simplistic rule-based algorithms by finding nonlinear relationships between variables and by spotting subtle clinical information which might go undetected by clinicians [12].

The aim of this study was to explore the ability of ML models to increase the number of chest pain patients accurately identified for rule-in or rule-out based on only the first blood tests after patient arrival, and to compare these models with the established 0 h arm of the ESC algorithm.

## Methods

### Study sites and design

This retrospective study included chest pain patients at the EDs of Skåne University Hospital at Lund (serving 320.000 inhabitants) and Helsingborg Hospital (serving 250.000 inhabitants) in Sweden. The aim of the study was to develop several combined diagnostic and prognostic tests of increasing complexity and to compare these to a baseline test using a single hs-cTnT value with prespecified cutoffs.

### Patient population

**Fig. 1:**
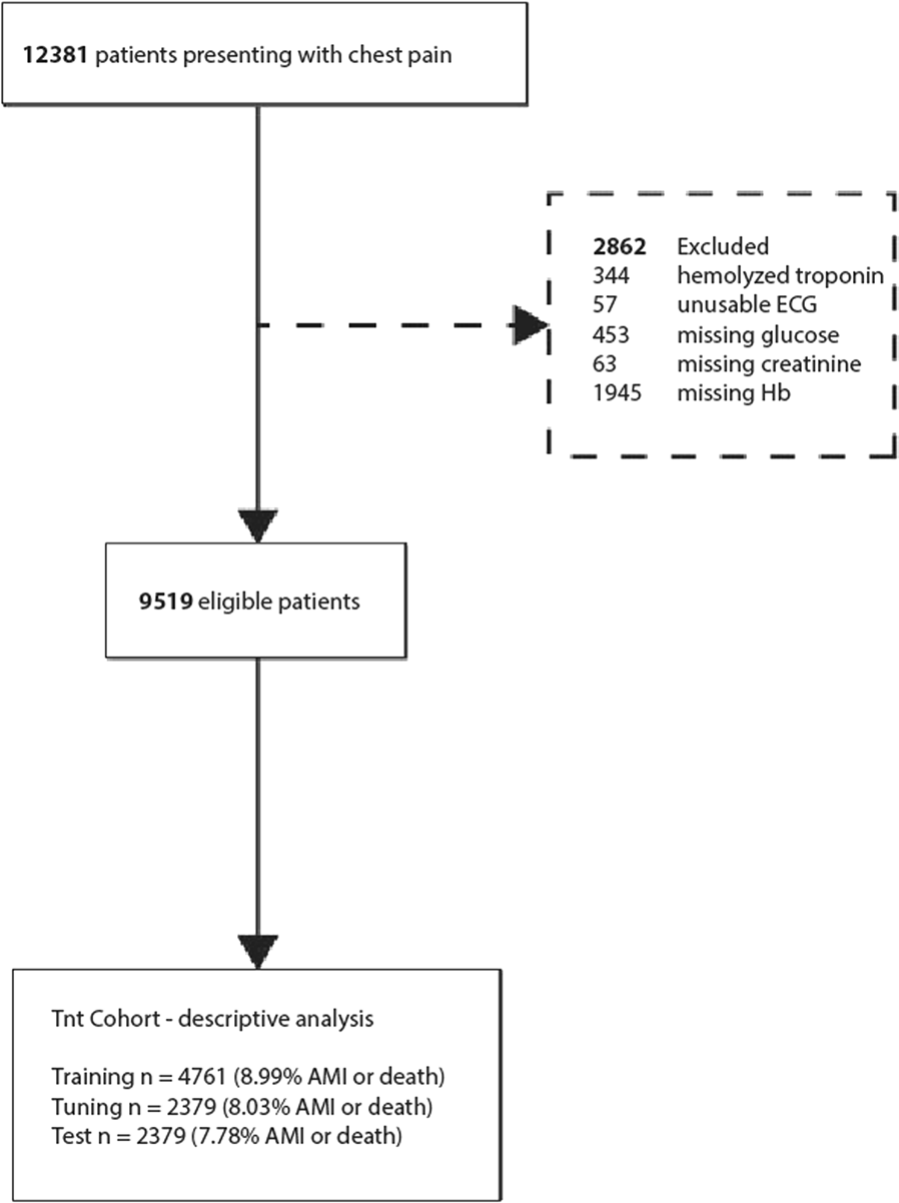
Flowchart of the study patients. n, number; ECG, electrocardiogram; Hb, Hemoglobin; AMI, acute myocardial infarction

This study utilized the EXPECT (Evaluation of Unknown Predictors of Electrocardiographic Changes – a Transnational Study) database [13, 14]. All adult patients (≥ 18 years) who presented with chest pain at the two EDs during 2013 and 2014 and had both hs-cTnT analyzed and electrocardiogram (ECG) recorded were included in the study. If a patient had multiple ED visits during this period, only the first was considered. Patients were excluded if hs-cTnT, glucose, creatinine or hemoglobin results at presentation were hemolyzed or missing, or if the ECG-signal was of low technical quality (Fig. 1).

For model training and evaluation, patients were chronologically split into three groups. The first 50% of patients were used to train the models, the following 25% of patients to further tune the models, i.e. to find optimal cut-offs for rule-out and rule-in while maintaining the prespecified sensitivity, NPV, specificity, and PPV thresholds (below). The final 25% of patients were used as a held-out testing group, to test model performance and verify that the performance thresholds were still reached. A timeline and distribution of these subsets during the study period can be found in Additional file 1: Fig. A4.

### Outcome measures

The primary clinical endpoint used in the evaluation was AMI or all-cause death within 30 days including the index visit. AMI during the index visit was a secondary clinical endpoint. The goal in this study was primarily to create diagnostic models. In an effort to not mislabel patients where the correct diagnosis was missed during the index event, 30 days was selected as a reasonable timeframe to obtain data on diagnoses.

The two primary outcome measures were (i) the size of the rule-out group on the condition that sensitivity for the endpoint was at least 99% and the negative predictive value (NPV) at least 99.5% and (ii) the size of the rule-in group provided that specificity was at least 90% and positive predictive value (PPV) at least 70%.

The AMI diagnoses were obtained from the regional patient records as ICD-10 diagnoses and death within 30 days from the Swedish population register. Index visit AMI was defined as a recorded diagnosis during the hospital admission directly following the ED visit. The diagnosis of AMI was made by the responsible physician as in routine care based on the diagnostic criteria for AMI and clinical judgment, either at the ED or, in case of admission, at the ward. ICD-10 diagnoses of AMI were taken from the hospital discharge records, and lacked more specific timestamps. At the time of the study, AMI was defined according to the third universal definition of myocardial infarction [15] as a rise/fall of hs-cTnT with at least one value above 14 ng/L with either symptoms suggestive of AMI, ECG changes, or imaging evidence of infarction. At the time of diagnosis, the responsible physician had access to patient records, ECG data, and blood samples, including serial hs-cTnT values. Validation of the ICD-10 diagnoses in the present study has been made previously, where overall agreement with expert physician adjudicators was 97% [16].

### ML input variables

As inputs to the machine learning models we used age, sex, ECG and the results of the first blood samples drawn after patient presentation to the ED; hs-cTnT, glucose, hemoglobin and creatinine. The selection of these variables was based on results from prior studies [9, 10, 17–20], as well as their widespread availability in different EDs.

All blood samples and ECG data were collected within 240 min of ED arrival.

### Blood sample analyses

Glucose was measured using Cobas 6000 (Roche Diagnostics) or with a spectrophotometric method using Radiometer ABL 800 flex Blood Gas Analyzer which uses the hexokinase method on serum. Hemoglobin (Hb) was measured with a spectrophotometry method using Radiometer ABL 800 flex Blood Gas Analyzer or using the Sysmex XN-10, using a spectrophotometric method on hemolyzed blood. Creatinine was analyzed using Cobas 6000 (Roche Diagnostics) or with a spectrophotometric method using Radiometer ABL 800 flex Blood Gas Analyzer. Details on analytical and reference ranges for these analyses can be found in the Additional file 1.

Samples of hs-cTnT were collected in lithium heparin tubes and analyzed with the Roche Cobas e602 (Roche Diagnostics). This assay has a limit of detection of 5 ng/L and a limit of blank of 3 ng/L. Coefficient of variation is < 10% at 13 ng/L and the 99^th^ percentile cut-off point is at 14 ng/L [21].

### ECG processing

When multiple ECGs were registered at the ED, we chose the one closest in time to the hs-cTnT as the most relevant for analysis. The 12-lead ECGs were 10 s long with a sample rate of 1000 Hz.

The Glasgow algorithm [22] was used to filter out ECG recordings of low technical quality. The algorithm also computes median beats (1.2 s) as well as numerous measures such as wave durations and amplitudes. Both the median beat and the raw signal were evaluated in the ML models.

### Machine learning models

**Fig. 2:**
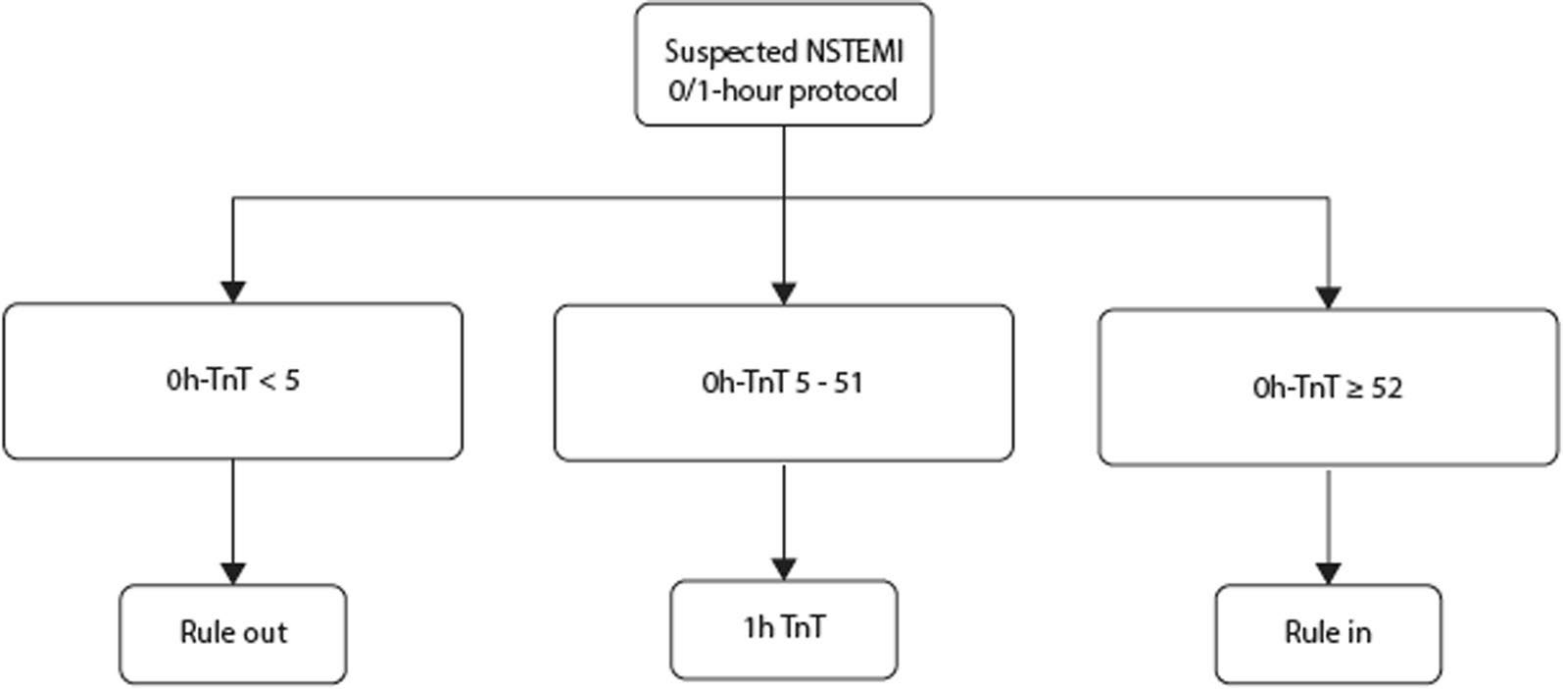
The 0 h arm of the European Society of Cardiology 0/1 h algorithm. NSTEMI, non ST-elevation myocardial infarction; TnT, Troponin T

We developed several different models for prediction. All models were developed using the same training data, but input variables differed among models. All ML models were compared to hs-cTnT alone as specified in the 0 h arm of the 0/1 h European Society of Cardiology (ESC) protocol (below denoted ESC 0 h) which is shown in Fig. 2. Specifically, the 0 h arm states that patients can be ruled out if 0 h hs-cTnT is < 5 ng/L, and ruled in if 0 h hs-cTnT is ≥ 52 ng/L. Patients with 5–51 ng/L are placed in an intermediate group [5] and require further evaluation such as additional hs-cTnT samples as implied by the 1 h arm of the 0/1 h ESC protocol, and/or cardiac imaging.

Logistic regression without interaction terms can be regarded as a special case of neural networks without any hidden layers. A logistic regression (denoted LogReg) model was thus implemented as a trivial neural network and trained using gradient descent until convergence. The inputs provided were age, sex, and the four blood test results. Additionally, a neural network model using the same inputs with one hidden layer (comprising 10 neurons) was also developed (ANN). In contrast to the LogReg model, the hidden layer in the ANN model enables it to learn interactions between the various inputs. As it uses the exact same inputs, the difference between the two models signifies the added value of considering more complex modeling, including interactions between the inputs.

To introduce the ECG signals, convolutional neural networks (CNN) were used. Two such models were built—one using the raw ECG signal (CNN-Raw) and one using the median beat (CNN-MB). In addition to the ECG, these models had access to the same inputs as the abovementioned models, i.e., age, sex, and the four biomarkers.

For each model, we selected two probability cutoffs. All patients below the lower probability were designated as rule-out, and all patients above the higher probability were designated as rule-in. Models were trained on the training set, and cutoffs were tuned on the tuning set. We then selected the cutoffs where the largest number of patients could be selected for rule-out and rule-in while still maintaining the target sensitivity, NPV, and PPV.

### Statistical analysis

Both the CNN and LogReg models give a probability between 0 and 1 as output for the chosen outcome. These models were evaluated using the area under the receiver operating characteristic curve (AUROC).

Continuous variables were described by mean and standard deviation or median and interquartile range, while categorical variables were described using proportions. For all rule-in and rule-out tests, sensitivity, specificity, PPV or NPV were calculated.

Independent samples T-tests were used for comparisons of continuous variables, while Pearson’s Chi-squared or Fisher's exact test were used for categorical variables. A p-value < 0.05 was considered statistically significant.

Bootstrapping with 1000 resamplings of the dataset was used to obtain 95% confidence intervals for the percentages of rule-in and rule-out for all models.

Models were created using the Python programming language (Python Software Foundation, Wilmington, Delaware, USA) and Tensorflow (Google LLC, Mountain View, California, USA).

## Results

### Patient characteristics

As detailed in Fig. 1, 12,381 patients were assessed for enrolment in the study. 2862 patients were excluded based on prespecified criteria, leaving 9519 patients in the final analysis. Excluded patients were less likely to have an AMI (7.3% vs 8.4%) (cf. Additional file 1: Table A1). As can be seen in Table 1, the mean age of the included patients was 59 years and 47.3% were female, and 804 (8.4%) patients had AMI or died within 30 days. Of these, 707 (88%) patients had an AMI during the index event. Patients with 30-day AMI or death were older (71.7 vs 57.9 years), more likely to be male and more often had prior diseases such as AMI, diabetes, or congestive heart failure. These patients also had higher blood hs-cTnT, glucose and creatinine levels, but Hb values were similar between patients with or without 30-day AMI or death.

**Table 1:** Characteristics of the included patients

	Total	30d AMI or death	Neither AMI nor death	p-values***
n (%)	9519 (100.0)	804 (8.4)	8715 (91.6)	
Female, %	47.3	35.1	48.4	< 0.01
Age, mean (std)	59.1 (18.9)	71.7 (12.9)	57.9 (18.9)	< 0.01
Disease history*				
Acute myocardial infarction, %	11.1	19.7	10.3	< 0.01
Congestive heart failure, %	8.6	14.9	8.0	< 0.01
Peripheral vascular disease, %	3.7	7.8	3.4	< 0.01
Cerebral vascular accident, %	6.4	8.8	6.2	< 0.01
Dementia, %	0.9	1.2	0.8	0.205
Pulmonary disease, %	11.3	11.3	11.2	0.95
Connective tissue disorder, %	2.9	3.9	2.8	0.095
Liver disease, %	0.2	0.4	0.2	0.426
Diabetes, %	10.7	17.8	10.1	< 0.01
Diabetes complications, %	4.5	9.2	4.1	< 0.01
Renal Disease, %	3.2	6.2	2.9	< 0.01
Cancer, %	8.4	12.7	8.0	< 0.01
Metastatic cancer, %	1.4	2.6	1.3	< 0.01
Severe liver disease, %	0.1	0.1	0.1	0.586
*Biomarkers*				
Glucose mmol/L, median (IQR)	6.1 (5.5–7.2)	7.2 (6.1–9)	6.1 (5.5–7.0)	< 0.01
Hb g/L, mean (std)	139.4 (16.4)	138.2 (20.1)	139.5 (16.0)	0.064
Creatinine (μmol/L), median (IQR)	78 (66–92)	85.5 (71–105)	77 (66–91)	< 0.001
hs-cTnT (ng/L), median (IQR)	6 (4–15)	52 (22–159)	6 (4–12)	< 0.001
hs-cTnT sample time, median (IQR)**	30.0 (17.0–49.0)	20.0 (10.0–36.0)	30.0 (17.0–50.0)	< 0.001

n, number; AMI, acute myocardial infarction; std, standard deviation; Hb, hemoglobin; hs-cTnT, high sensitivity cardiac troponin T; IQR, interquartile range

*As recorded up to 5 years prior to study event

**Time from patient presentation to sampling of hs-cTnT

***p-values for differences between the group with 30d AMI or Death, and the group with neither AMI nor Death

The median time from patient arrival in the emergency department to hs-cTnT sampling was 30 min for all patients and 20 min for patients with 30-day AMI or death.

Of the 9519 patients, 2379 (25%) were put aside as the testing group and 2379 as a tuning group. The remaining 4761 (50%) patients formed the training group.

The prevalence of 30-day AMI/death was 9.0% in the training group, 8.0% in the tuning group and 7.8% in the testing group (Additional file 1: Table A6). The tuning and testing groups had similar distributions of age, sex, and comorbidities. The training group were slightly older (60.3 vs 57.4), and had more comorbidities. A larger portion of patients in the testing group had hs-cTnT < 5 ng/L (47.2%) than in the tuning group (35.8%).

### Main results

The models were evaluated both in the testing group (Table 2) and the tuning group (Additional file 1: Table A2). AUROC values in these groups are provided in Additional file 1: Table A3.

**Table 2:** Comparison of methods—testing set

	Sens (95% CI)	NPV (95% CI)	Ruled out % (95% CI)	Ruled out (n)	Missed AMI or Death
Rule out					
ESC 0 h	98.9 (96.15–99.87)	99.8 (99.30–99.96)	47.2 (45.1–49.3)	1123	2
LogReg	97.8 (94.56–99.41)	99.6 (98.85–99.83)	38.5 (36.5–40.4)	915	4
ANN	99.5 (97.03–99.99)	99.9 (99.37–99.99)	46.6 (44.6–48.7)	1109	1
CNN-MB	99.5 (97.03–99.99)	99.9 (99.46–99.99)	55 (53.1–57)	1309	1
CNN-RAW	99.5 (97.03–99.99)	99.9 (99.42–99.99)	50.8 (48.8–52.9)	1208	1

	Spec (95% CI)	PPV (95% CI)	Ruled in % (95% CI)	Ruled in (n)	Incorrectly ruled in
Rule in					
ESC 0 h	97.4 (96.65–98.03)	63.9 (57.06–70.27)	6.6 (5.7–7.8)	158	57
LogReg	98.4 (97.74–98.85)	65.1 (56.09–73.05)	4.3 (3.5–5.2)	103	36
ANN	98.2 (97.58–98.73)	69.8 (62.05–76.51)	5.4 (4.5–6.4)	129	39
CNN-MB	98.5 (97.89–98.96)	73.6 (65.86–80.12)	5.3 (4.4–6.2)	125	33
CNN-RAW	98.2 (97.58–98.73)	70.5 (62.87–77.06)	5.5 (4.6–6.5)	132	39

Performance with respect to rule-out (sensitivity and NPV) and rule-in (Specificity and PPV)

NPV, negative predictive value; PPV, positive predictive value; n, number; ESC 0 h, 0 h arm of the European Society of Cardiology algorithm; LogReg, logistic regression; ANN, artificial neural network, CNN-MB, convolutional neural network trained on median beat ECG data; CNN-RAW, convolutional neural network trained on raw ECG data

**Table 3:** Comparison of ESC 0 h and CNN-MB

	CNN-MB-RuleOut	CNN-MB-Intermediate	CNN-MB-RuleIn
Patients with 30d AMI or death (n = 185)			
ESC 0 h-RuleOut	1	1	0
ESC 0 h-Intermediate	0	81	1
ESC 0 h-RuleIn	0	10	91
Patients without 30d AMI or death (n = 2194)			
ESC 0 h-RuleOut	1035	86	0
ESC 0 h-Intermediate	273	738	5
ESC 0 h-RuleIn	0	29	28

Patients with or without 30d AMI or death are separated into two halves, and the number of patients ending up in the different groups according to ESC 0 h and CNN-MB are indicated by rows and columns. Numbers along the diagonal indicate that the models agree

AMI, Acute myocardial infarction; ESC 0 h, 0 h arm of the European Society of Cardiology algorithm; CNN-MB, convolutional neural network trained on median beat ECG data

#### ESC 0 h versus machine learning models: rule-out

As can be seen in Table 2, in the testing group ESC 0 h (< 5 ng/L) identified 1123 (47.2%) patients for rule-out, with a 98.9% sensitivity and 99.8% NPV for AMI or death within 30 days. The LogReg model had an AUROC of 86.4 and, at a sensitivity of 97.8% and NPV of 99.6%, it identified fewer patients for rule-out than ESC 0 h; 915 (38.5%). With the simple ANN, the AUROC increased to 91.9 and the number of ruled-out patients increased to 1109 (46.6%). The ML models that included the ECG performed best. CNN-RAW had an AUROC of 93.8 and ruled out 1208 patients (50.8%), and CNN-MB had an AUROC of 93.9 and ruled out 1309 (55.0%) patients. Notably, the 95% CIs between ESC 0 h (45.1–49.3) and CNN MB (53.1–57) did not overlap. This shows that there was a significant difference in the amount of patients ruled out by the models.

The results in the tuning group can be seen in Additional file 1: Table A2. In these patients, ESC 0 h and the ML models selected fewer patients for rule-out compared to the testing groups, but all models had higher rule-out percentage than ESC 0 h. Most importantly, the relative order among models remained the same between both testing and tuning groups.

#### ESC 0 h versus machine learning models: rule-in

As seen in Table 2, in the testing group, ESC 0 h (≥ 52 ng/L) ruled in 158 patients (6.6%) at a specificity of 97.4% and a PPV of 63.9%. ESC 0 h thereby ruled in the most patients of all tested models, but it did not reach our prespecified PPV of 70%. Among the generated models, performance differences at our specificity and PPV thresholds were small, with LogReg ruling in the fewest patients (103; 4.3%), and CNN-RAW the most (132, 5.5%). Both CNN models maintained a PPV above 70%, but the LogReg and ANN models failed to reach this target. For all models, the number of patients ruled in was similar in the testing and tuning groups.

#### Combined rule-out and rule-in

ESC 0 h selected 1020 patients (42.9%) for either rule-in or rule-out in the tuning group, and 1281 patients (53.8%) in the testing group.

Among the generated models, the CNN-MB model identified the largest total number of patients for either rule-out or rule-in in both the tuning (1373, 57.7%) and testing (1434, 60%) sets, while maintaining our sensitivity, NPV, specificity and PPV requirements.

#### Performance on index-visit AMI

As the 0/1 h ESC algorithm was initially developed for index visit AMI, models were also compared using this endpoint. Model performance was similar, but only the CNN-MB model achieved a PPV over 70%. See Additional file 1: Table A7 for further details.

#### Comparison on patient level

**Fig. 3:**
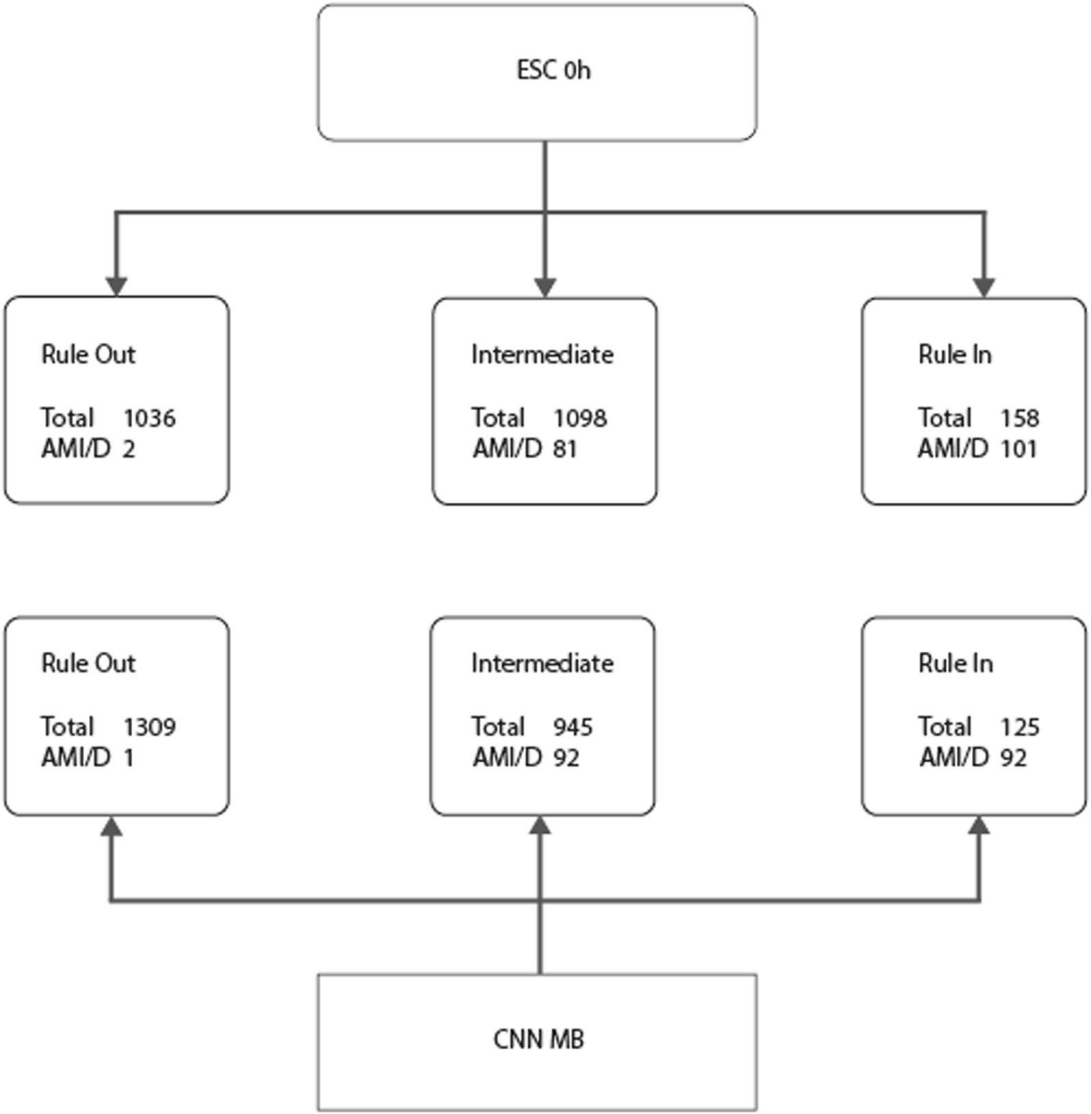
Comparison between the 0 h arm of the European Society of Cardiology 0/1 h algorithm (ESC 0 h) and the CNN-MB model. AMI/D, 30 day AMI or death

To further understand the performance difference between the rule-based ESC 0 h and ML models, we compared rule-out with ESC 0 h and CNN-MB in the testing group, as shown in Table 3 and Fig. 3. Among patients with 30-day AMI or death, 10 patients were ruled in by ESC 0 h while CNN-MB placed them in the intermediate group. Conversely, for patients without 30-day AMI or death, ESC 0 h ruled in 57 patients, which explains its low PPV. Further, ESC 0 h put 273 patients without AMI or 30-day death in the intermediate group, all of whom were ruled out by CNN-MB. This is the main explanation why the rule-out group size was markedly larger with the CNN-MB than ESC 0 h. Of the 1309 patients ruled out by CNN-MB, 1036 patients (79.1%) would have been ruled out by ESC 0 h.

## Discussion

In this study we compared different strategies combining patient age, sex, ECG data and hs-cTnT, glucose, hemoglobin and creatinine levels at patient arrival for rule-in and rule-out of 30-day AMI or death. We made three major findings.

*First*, using a CNN model, 30-day AMI or death could be safely ruled out in over 50% of patients with the use of only one troponin test together with other biomarkers commonly used in routine care, and the ECG. This has the potential to decrease the number of blood tests and length of ED stay for these patients, and may also help to reduce ED crowding. 79% of these patients were also ruled out by the ESC 0 h rule. *Second*, the CNN model identified around 5% of patients for rule-in, and an early decision to admit these patients might be warranted. *Third*, increased complexity in model architecture and inputs gave improved performance. Our ANN model identified more patients for rule-in or rule-out than the logistic regression model using the same inputs. Similarly, extending the ANN model with the ECG (i.e., the CNN models) led to a further improvement.

Previous studies have shown that an ML model using *two serial* troponins, age and sex may outperform the traditional ESC 0/3 h pathway [23]. Our study expanded on these results by using *a single initial* troponin test in combination with other biomarkers and the ECG in order to further reduce the time from patient arrival to clinical decision.

Several studies have combined rule-based algorithms with single or serial negative troponin tests for rule-out, and shown that this increases the sensitivity and NPV, but decreases the fraction of patients identified for rule-out, compared to using only troponin tests [4, 24–26]. Our results suggest that ML models could be used to rule out AMI or death in a larger proportion of patients while still maintaining high sensitivity and NPV values. The size of the rule-out group in our study was comparable or larger than in similar studies using additional hs-cTnT samples [27].

There are some considerations with the use of complex ML models as decision support. The models’ complexity traditionally comes at the cost of decreased explainability, i.e. that it is harder for the user to understand the basis of the predictions. To maintain user trust, this disadvantage may necessitate a significantly improved predictive performance with the ML model [28, 29]. In the future, ML models for use in the medical field might be required by law to be reasonably explainable [30], and efforts are ongoing to achieve this [31].

### Limitations

Since input data to the models included troponin and ECG, which were also part of the diagnostic criteria for AMI, there was a risk of incorporation bias. Since all our models included troponin, which by far is the most important diagnostic factor for AMI, we find it reasonable to believe that any such bias would affect all models to a similar degree, and thus not qualitatively change the results.

There were few patients with 30-day AMI or death in the tuning and testing groups. To attain our 99% threshold for sensitivity, a maximum of one patient with 30-day AMI or death was allowed among the patients ruled out (as a false negative). This caused the models to be conservative, as a single outlier could reduce the rule-out threshold for the entire model. The percentage of patients identified for rule-out may thus be higher in populations with more events.

Using ICD codes for the AMI diagnoses may have led to mislabeling of patients, as these codes might be incorrect. There was no adjudication of the diagnoses in our study, which is a limitation. However, using ICD codes does reflect clinical reality, and a comparison with adjudicated diagnoses from a parallel study showed an agreement of 97% [16]. Also, it seems reasonable to believe that a few misclassified cases will affect all models similarly, and that the ranking of the models will remain the same.

The mean hs-cTnT value and the number of patients with hs-cTnT < 5 ng/L in the tuning and testing sets differed somewhat, which was notable as the percentage of patients with 30-day AMI or death were similar. We suspect that this could be due to a more frequent troponin sampling during the later parts of the study period, thus including more patients with lower hs-cTnT levels, where hs-cTnT tests previously would not have been ordered. There were no changes in the hs-cTnT assay during the study period.

We did not have data on the time from chest pain onset to hs-cTnT sampling, and thus included all patients, regardless of this time. Some AMI patients presenting early might thereby have been falsely ruled out, reducing model performance. In the present study, excluding early presenters (e.g. within 3 h of chest pain onset) may have resulted in higher sensitivities and PPV values, possibly at the cost of ruling out fewer patients. Again, this affects all models equally and the main conclusion likely remains valid.

It should also be noted that our results might not be generalizable to other settings and populations. Compared to other cohorts, our patients might be younger and more often female. However, it seems reasonable to believe that the relative performance of the algorithms would be similar in other populations. Before clinical implementation, any ML model should be externally validated in independent cohorts and prospectively tested in the specific healthcare setting.

## Conclusion

In ED chest pain patients, a CNN based on patient age, sex, ECG, and the first blood tests at patient presentation for hs-cTnT, glucose, creatinine, and hemoglobin, was able to identify a total of 60% of the patients for safe and early rule-in or rule-out of 30-day AMI or death. A decision support system based on such a CNN has the potential to reduce the number of blood tests and decrease the length of ED stay for chest pain patients, and to help decrease ED crowding.

Model performance and safety was the focus of this study, and the results should now be validated prospectively, ideally in randomized trials at multiple centers. However, before implementation of these decision aids in routine care, issues regarding transparency, accountability, and user acceptance should also be considered.

## Supplementary Information

**Additional file 1.** Appendix.

## Data Availability

The datasets analysed during the current study are not publicly available due to data privacy reasons, but are available from the corresponding author on reasonable request. The authors are willing to apply models from external researchers on the same data set upon request, assuming the models can be provided.

## Abbreviations

AUROC: Area under the Receiver Operating Curve
AMI: Acute Myocardial Infarction
ANN: Artificial Neural Network
ACS: Acute coronary syndrome
LOG-REG: Logistic regression
CNN: Convolutional Neural Network
CNN-MB: Convolutional neural network, median beat ecg data
CNN-RAW: Convolutional neural network, raw ecg data
hs-cTnT: High-sensitivity Troponin T
ECG: Electrocardiogram
STEMI: ST elevation myocardial infarction
ESC 0h: 0H arm of the European Society of Cardiology algorithm
CI: Confidence interval

## References

[1] Hollander JE (1999). The continuing search to identify the very-low-risk chest pain patient. Acad Emerg Med.

[2] Eriksson D, Khoshnood A, Larsson D, Lundager-Forberg J, Mokhtari A, Ekelund U (2019). Diagnostic accuracy of history and physical examination for predicting major adverse cardiac events within 30 days in patients with acute chest pain. J Emerg Med.

[3] Mokhtari A, Dryver E, Söderholm M, Ekelund U (2015). Diagnostic values of chest pain history, ECG, troponin and clinical gestalt in patients with chest pain and potential acute coronary syndrome assessed in the emergency department. Springerplus.

[4] Nilsson T, Lundberg G, Larsson D, Mokhtari A, Ekelund U (2020). Emergency department chest pain patients with or without ongoing pain: characteristics, outcome, and diagnostic value of the electrocardiogram. J Emerg Med.

[5] Collet JP, Thiele H, Barbato E (2020). ESC Guidelines for the management of acute coronary syndromes in patients presenting without persistent ST-segment elevation. Eur Heart J.

[6] Neumann JT, Twerenbold R, Ojeda F (2019). Application of high-sensitivity troponin in suspected myocardial infarction. N Engl J Med.

[7] Aviles RJ, Askari AT, Lindahl B (2002). Troponin T levels in patients with acute coronary syndromes, with or without renal dysfunction. N Engl J Med.

[8] Sarnak MJ, Levey AS (2000). Cardiovascular disease and chronic renal disease: a new paradigm. Am J Kidney Dis.

[9] Feng QZ, Zhao YS, Li YF (2011). Effect of haemoglobin concentration on the clinical outcomes in patients with acute myocardial infarction and the factors related to haemoglobin. BMC Res Notes.

[10] Olsson P, Khoshnood A, Mokhtari A, Ekelund U (2021). Glucose and high-sensitivity troponin T predict a low risk of major adverse cardiac events in emergency department chest pain patients. Scand Cardiovasc J.

[11] Furlong JW, Dupuy ME, Heinsimer JA (1991). Neural network analysis of serial cardiac enzyme data. A clinical application of artificial machine intelligence. Am J Clin Pathol.

[12] Miotto R, Li L, Kidd BA, Dudley JT (2016). Deep patient: an unsupervised representation to predict the future of patients from the electronic health records. Sci Rep.

[13] Hansen TG, Pottegård A, Brandes A (2020). New-onset atrial fibrillation among patients with infection in the emergency department: a multicenter cohort study of 1-year stroke risk. Am J Med.

[14] Schade Hansen C, Pottegård A, Ekelund U (2018). Association between QTc prolongation and mortality in patients with suspected poisoning in the emergency department: a transnational propensity score matched cohort study. BMJ Open.

[15] Thygesen K, Alpert JS, Jaffe AS, Simoons ML, Chaitman BR, White HD (2012). Third universal definition of myocardial infarction. Circulation.

[16] Björkelund A, Ohlsson M, Lundager Forberg J (2021). Machine learning compared with rule-in/rule-out algorithms and logistic regression to predict acute myocardial infarction based on troponin T concentrations. J Am Coll Emerg Phys Open.

[17] Greenslade JH, Kavsak P, Parsonage W (2015). Combining presentation high-sensitivity cardiac troponin I and glucose measurements to rule-out an acute myocardial infarction in patients presenting to emergency department with chest pain. Clin Biochem.

[18] Shortt C, Ma J, Clayton N (2017). Rule-in and rule-out of myocardial infarction using cardiac troponin and glycemic biomarkers in patients with symptoms suggestive of acute coronary syndrome. Clin Chem.

[19] Haller PM, Neumann JT, Sörensen NA (2021). The association of anaemia and high-sensitivity cardiac troponin and its effect on diagnosing myocardial infarction. Eur Heart J Acute Cardiovasc Care.

[20] Kavsak P, Neumann J, Cullen L (2018). Clinical chemistry score versus high-sensitivity cardiac troponin I and T tests alone to identify patients at low or high risk for myocardial infarction or death at presentation to the emergency department. Can Med Assoc J.

[21] Giannitsis E, Kurz K, Hallermayer K, Jarausch J, Jaffe AS, Katus HA (2010). Analytical validation of a high-sensitivity cardiac troponin T assay. Clin Chem.

[22] Macfarlane PW, Devine B, Clark E. The University of Glasgow (Uni-G) ECG Analysis Program, vol 32. 2005. 10.1109/CIC.2005.1588134.

[23] Than MP, Pickering JW, Sandoval Y (2019). Machine learning to predict the likelihood of acute myocardial infarction. Circulation.

[24] Mokhtari A, Borna C, Gilje P (2016). A 1-h combination algorithm allows fast rule-out and rule-in of major adverse cardiac events. J Am Coll Cardiol.

[25] Khoshnood A, Erlandsson M, Isma N, Yndigegn T, Mokhtari A (2020). Diagnostic accuracy of troponin T measured ≥6h after symptom onset for ruling out myocardial infarction. Scand Cardiovasc J SCJ.

[26] Mokhtari A, Lindahl B, Smith JG, Holzmann MJ, Khoshnood A, Ekelund U (2016). Diagnostic accuracy of high-sensitivity cardiac troponin T at presentation combined with history and ECG for ruling out major adverse cardiac events. Ann Emerg Med.

[27] Mokhtari A, Lindahl B, Schiopu A (2017). A 0-hour/1-hour protocol for safe, early discharge of chest pain patients. Diercks DB, ed. Acad Emerg Med.

[28] Holzinger A, Biemann C, Pattichis CS, Kell DB. What do we need to build explainable AI systems for the medical domain? arXiv. https://arxiv.org/abs/1712.09923. Accessed 16 Mar 2021.

[29] Samek W, Wiegand T, Müller KR. Explainable artificial intelligence: understanding, visualizing and interpreting deep learning models. arXiv. 2017. Accessed 16 Mar 2021. https://arxiv.org/abs/1708.08296

[30] Hacker P, Krestel R, Grundmann S, Naumann F (2020). Explainable AI under contract and tort law: legal incentives and technical challenges. Artif Intell Law.

[31] Vilone G, Longo L. Explainable artificial intelligence: a systematic review. CoRR. 2020. https://arxiv.org/abs/2006.00093.

