# Supplementary material for: Machine learning for early prediction of acute myocardial infarction or death in acute chest pain patients using electrocardiogram and blood tests at presentation
Source: BMC Med Inform Decis Mak. 2023 Feb 2;23:25. doi: 10.1186/s12911-023-02119-1 (PMC9896766; doi:10.1186/s12911-023-02119-1)
Supplement: Supplementary file 1 — Additional file 1. Appendix. [file 12911_2023_2119_MOESM1_ESM.docx]

Appendix

**Analytical ranges**

**Glucose**

The Radiometer 800 Flex Analyzer had an analytical range of 0 - 60 mmol/L and reference range 3.89 - 5.83 mmol/L for glucose.
For COBAS 6000, glucose had a measuring range of 0.11 - 4.6 mmol/L and a reference range of 4.2 - 6.0 mmol/L. The limit of detection was 0.11 mmol/L.CV% was 1.0 at glucose levels of 3 mmol/L and 1.1 at levels of 20 mmol/L.

Local comparisons of results from the Radiometer 800 Flex Analyzer and COBAS 6000 machines demonstrated that the mean difference was less than 1%.

**Hemoglobin**

Hb measured on the Radiometer 800 Flex Analyzer had an analytical range of 0 - 277 g/L and a reference range of 135-175 g/L in men and 120-160 g/L in women.

Hemoglobin measured on the Sysmex XN-10 had a measurement range of 0 - 260 g/L and a reference range of 117-153 g/L for women and 134-170 g/L for men.

The limit of detection for hemoglobin was 2 g/L. CV% was 0.9 at hemoglobin levels of 59 g/L, 0.5 at levels of 125 g/L and 0.6 at levels of 168 g/L.

Local comparisons of the results from the Radiometer 800 Flex Analyzer and the Sysmex XN-10 demonstrated that differences were less than 10%.

**Creatinine**

Creatinine measured on the Radiometer 800 flex Analyzer had a limit of blank of 3.2 μmol/L and a limit of detection of 5.3 μmol/L. The measuring range is 10-1800 μmol/L. The reference range for creatinine was 53-106 μmol/L for men and 44-97 μmol/L for women as described by the radiometer 800 flex Analyzer manual.

Creatinine had a measuring range of 5 – 2 700 µmol/L using the COBAS 6000, and a reference range of 60 - 105 µmol/L for men and 45 - 90 µmol/L for women. The limit of detection was 5 µmol/L and the CV% was 1.4 for creatinine levels of 70 µmol/L and 1.7 for levels around 600 µmol/L [1 - 4].

A comparison between the two measuring instruments has been made locally in 2018. This showed a variation of < 10% between measurements taken on the COBAS 6000 and the Radiometer 800 Flex Analyzer machines.

**Missing variables**

Missingness was handled by excluding all cases with incomplete data. As this might introduce a bias we examined the relationship between missingness of a variable and the outcome of 30 day AMI or death. The results are presented in table A8. There was no significant difference between missingness of blood samples or ECG data, and the outcome variables.

**Details on deep learning models**

The logistic regression model (LogReg) was trained for 200 epochs using the Adam optimizer, learning rate 0.003 and a batch size of 32.

The CNN models that included the ECG used rectified linear units for activation and were trained with the Adam optimizer for 200 epochs with a learning rate of 0.003 and batch size 32. The ECG signal was fed through 1-dimensional convolutional layers followed by max pooling and dropout. Then, the convoluted signal was merged with the remaining inputs (age, sex, and blood samples), passed through another hidden layer, before final activation. The architectures of the two networks with further details on hyperparameters are shown in Figure A5.

The hyperparameters and structures of the networks were found using random search over the tuning set [5]. Specifically, (a) the number of layers for the various inputs (e.g., how many convolutional layers to use for the ECG, how many hidden layers after merging ECG with the other features), (b) the hyperparameters of those layers (e.g., kernel size of CNN layers, dropout rates, or number of neurons in a hidden layer), as well as (c) learning related parameters (e.g., batch sizes, optimization algorithm, or learning rate) were subject to random search. The parameters were sampled from reasonable distributions with respect to each type of parameter (e.g., the number of convolutional layers in the CNN case was drawn to be any integer between 1 and 10, whereas dropout rates were allowed to range between 0 and 1, etc). After hundreds of runs for model type, models were ranked by AUC on the tuning set and the best model was chosen. The top *k* models within 0.001 in AUC from the highest scoring model were considered tied for the best, and the tie was broken by selecting the model with the lowest training loss on the tuning set in the last epoch. Only 8 leads (I, II, and V1-V6) are used as inputs for the networks, since the other 4 merely are linear combinations of the others and thus do not contribute any more information.

For ANN and CNN models, an ensemble approach was used to reduce the potential noise from local optima. Specifically, five identical networks that differed only in their random initialization were trained for each case and their predictions combined by taking the mean predictions.

These models were trained and tested on register data, and as such have not been implemented in clinical practice. Digital access to blood sample data and ECGs will differ between hospitals, but once an adequate data feeding system is set up, models are very fast. A single model of the present types can classify thousands of patients within a minute on a standard computer.

| **Table A1: Excluded patients** | | | |
| --- | --- | --- | --- |
|  | Total | 30d AMI or Death | No AMI or Death |
| n (%) | 2862 (100.0) | 210 (7.3) | 2652 (92.7) |
| Female, % | 48.2 | 39 | 48.9 |
| Age, mean (std) | 59.6 (18.6) | 73.0 (12.8) | 58.5 (18.6) |
| **Disease history *** | | | |
| Acute Myocardial Infarction, % | 13.4 | 23.8 | 12.6 |
| Congestive Heart Failure, % | 11.2 | 19.5 | 10.6 |
| Peripheral Vascular Disease, % | 4.6 | 7.6 | 4.4 |
| Cerebral Vascular Accident, % | 7.4 | 10 | 7.2 |
| Dementia, % | 0.8 | 3.8 | 0.6 |
| Pulmonary Disease, % | 13.9 | 18.1 | 13.6 |
| Connective Tissue Disorder, % | 3.2 | 7.6 | 2.9 |
| Liver Disease, % | 0.4 | 0.5 | 0.4 |
| Diabetes, % | 12.2 | 22.4 | 11.4 |
| Diabetes Complications, % | 5.8 | 9 | 5.5 |
| Renal Disease, % | 3.7 | 7.1 | 3.4 |
| Cancer, % | 7.9 | 9.5 | 7.7 |
| Metastatic Cancer, % | 0.9 | 1 | 0.9 |
| Severe Liver Disease, % | 0.1 | 0 | 0.2 |
| **Biomarkers** | | | |
| Glukos mmol/L , median (iqr) | 6.1 (5.5 - 7.2) | 7.1 (6.1 - 8.5) | 6.1 (5.5 - 7.1) |
| Hb g/L, mean (std) | 139.8 (16.2) | 141.5 (20.3) | 139.6 (15.8) |
| Creatinine (μmol/L), median (iqr) | 82.0 (70.0 - 96.0) | 92.0 (78.0 - 113.8) | 81.0 (69.0 - 95.0) |
| TnT (ng/L), median (iqr) | 7.0 (4.0 - 15.0) | 54.0 (26.0 - 148.5) | 6.0 (4.0 - 12.0) |
| n, number; AMI, acute myocardial infarction; std, standard deviation;  iqr, Interquartile range; Hb, hemoglobin;  hs-cTnT, high sensitivity cardiac troponin T  * As recorded up to 5 years prior to study event. | | | |

| **Table A2: Extended Comparison of models - tuning set** | | | | | | | | |
| --- | --- | --- | --- | --- | --- | --- | --- | --- |
|  | **Rule-out** | | | | **Rule-in** | | | |
|  | **Sensitivity** | **NPV** | **Ruled out** | **(%)** | **Specificity** | **PPV** | **Ruled in** | **(%)** |
| ESC 0h | 100.0 | 100.0 | 851 | 35.8% | 96.9 | 60.4 | 169 | 7.1% |
| LogReg | 99.5 | 99.9 | 874 | 36.7% | 98.6 | 70.3 | 101 | 4.2% |
| ANN | 99.5 | 99.9 | 1043 | 43.8% | 98.2 | 70.4 | 135 | 5.7% |
| CNN-MB | 99.5 | 99.9 | 1233 | 51.8% | 98.1 | 70.0 | 140 | 5.9% |
| CNN-RAW | 99.5 | 99.9 | 1056 | 44.4% | 98.1 | 70.1 | 137 | 5.8% |
| Comparison of all models, including the additional ones from the sensitivity analysis. Models evaluated on the tuning set.  Performance with respect to rule-out (sensitivity and NPV) and rule-in (Specificity and PPV).  NPV, negative predictive value; PPV, positive predictive value;  ESC 0h, 0h arm of the European Society of Cardiology algorithm;  LogReg, logistic regression; ANN, artificial neural network; CNN-MB, convolutional neural network trained on median beat ECG data;  CNN-RAW, convolutional neural network trained on raw ECG data. | | | | | | | | |

| **Table A3: ROC AUC values** | | |
| --- | --- | --- |
| **Label** | **Tuning** | **testing** |
| LogReg | 87.2% | 86.4% |
| ANN | 91.9% | 91.9% |
| CNN-MB | 93.7% | 93.9% |
| CNN-RAW | 93.4% | 93.8% |
| Performance of individual models with respect to the area under the receiver operating curve for both tuning and testing sets. | | |


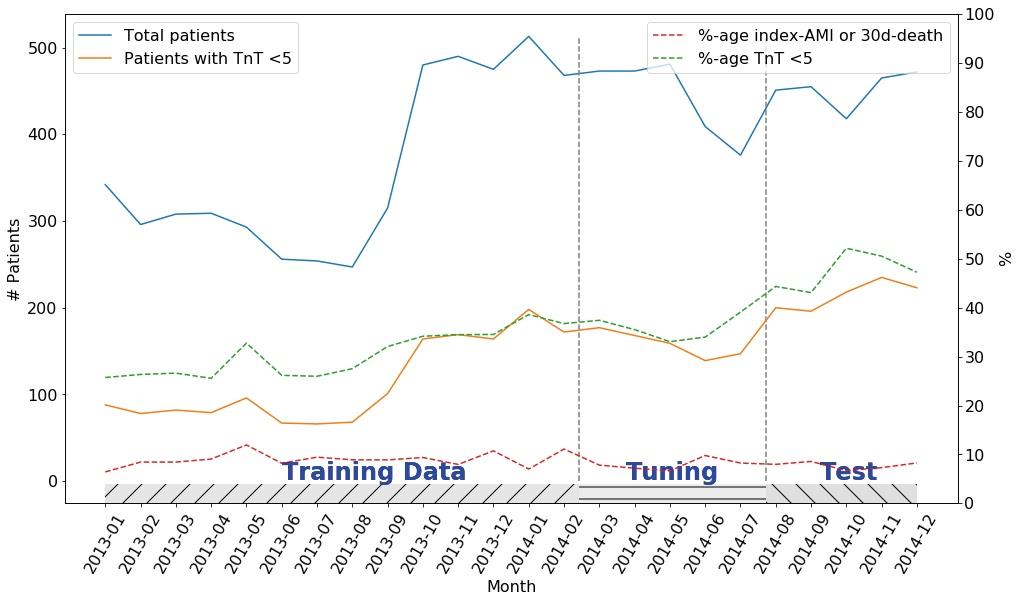


**Figure A4: Timeline of patients**. The total number of patients (blue) and those with hs-cTnT <5 (orange) are shown, as well as the percentages of those with hs-cTnT <5 (dashed green) or 30d AMI/death (red), broken down by month. The partition of patients into training, tuning, and testing groups is shown on the x-axis.


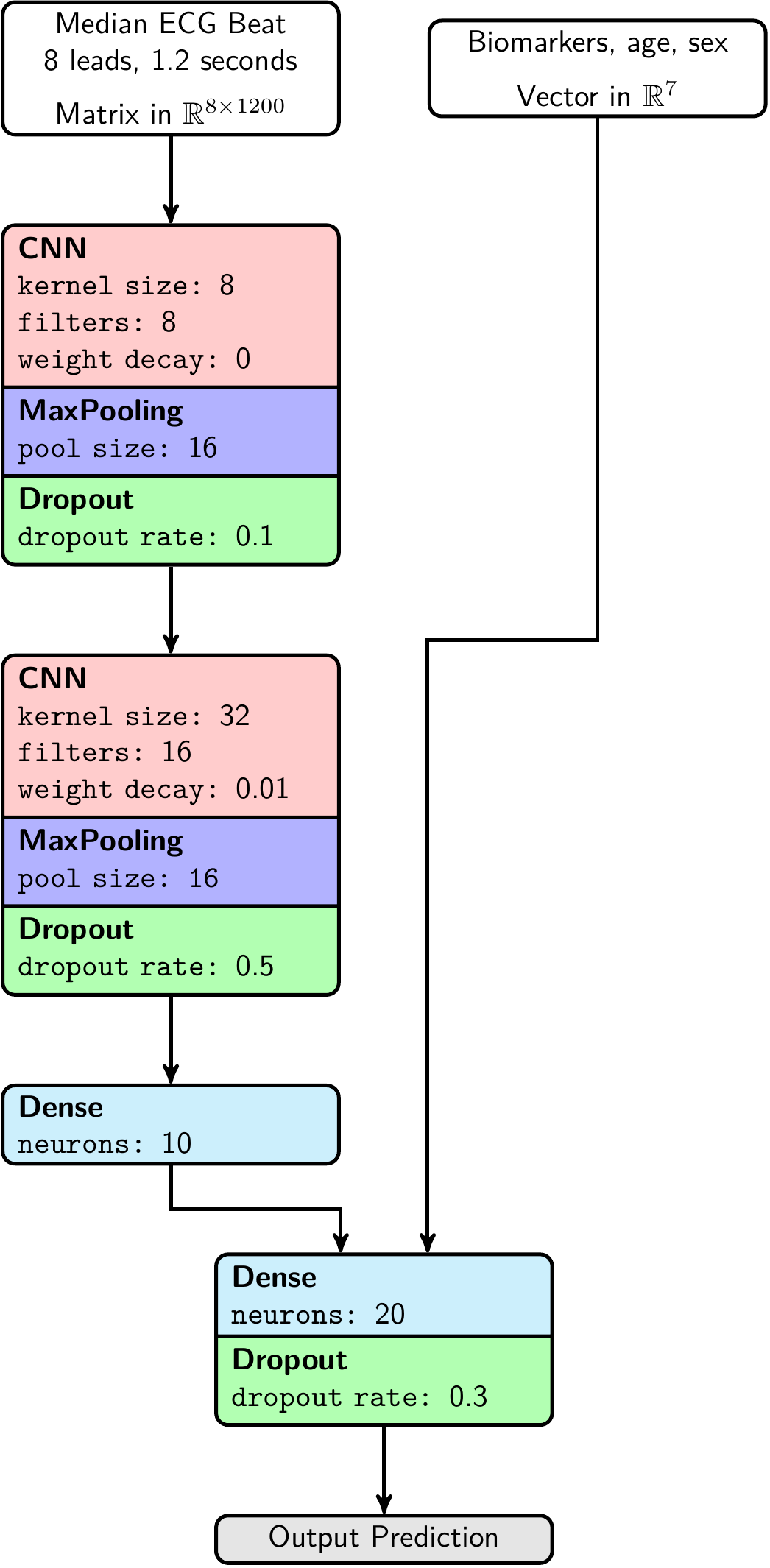

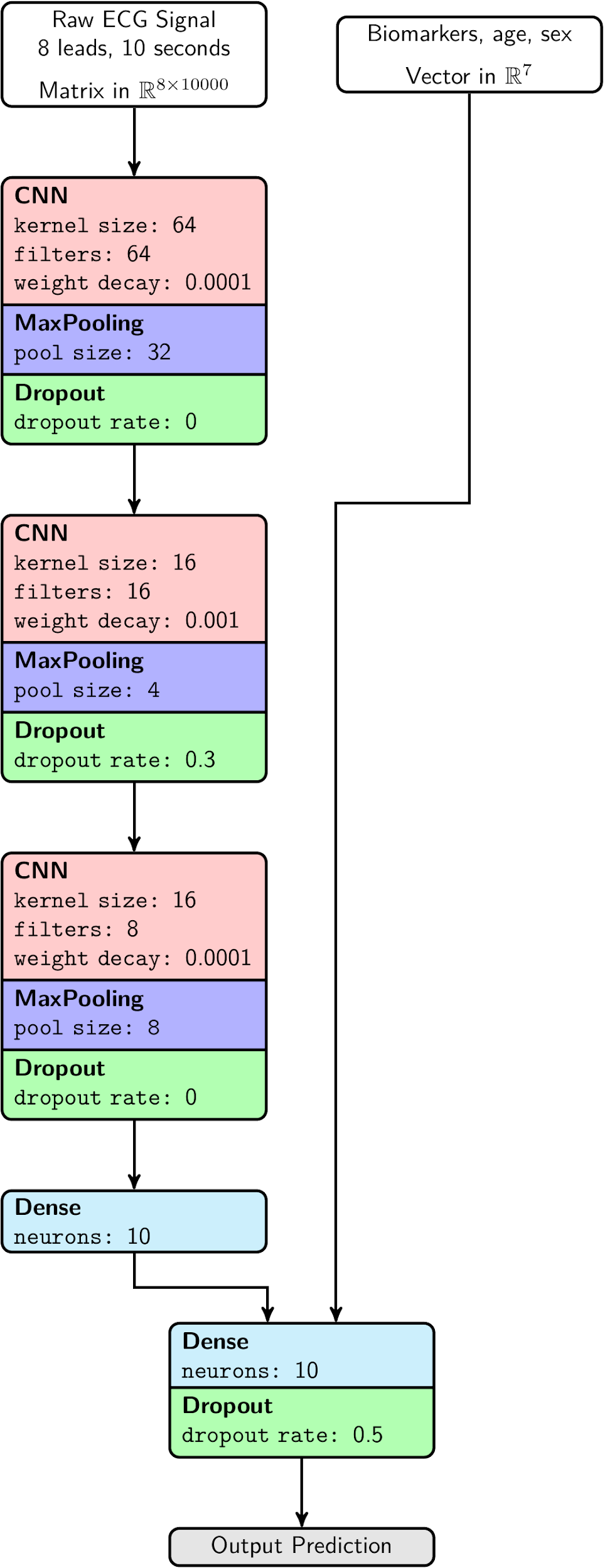
**Figure A5.** Network architectures for the convolutional neural networks using the median beat and raw signals, respectively.

| **Table A6 Patient subgroups** | | | |
| --- | --- | --- | --- |
|  | **train** | **tune** | **test** |
| n | 4761 | 2379 | 2379 |
| Female, % | 47.1 | 47.7 | 47.4 |
| Age, mean (std) | 60.3 (18.8) | 58.3 (19.0) | 57.4 (18.8) |
| AcuteMyocardialInfarction, % | 12.5 | 9.8 | 9.6 |
| CongestiveHeartFailure, % | 9.7 | 8.1 | 6.6 |
| PeripheralVascularDisease, % | 4.1 | 3.2 | 3.5 |
| CerebralVascularAccident, % | 7.1 | 5 | 6.2 |
| Dementia, % | 0.9 | 0.8 | 0.7 |
| PulmonaryDisease, % | 12.1 | 10.4 | 10.5 |
| ConnectiveTissueDisorder, % | 3.2 | 2.9 | 2.4 |
| LiverDisease, % | 0.3 | 0.1 | 0.2 |
| Diabetes, % | 12.5 | 9.4 | 8.7 |
| DiabetesComplications, % | 5.1 | 4 | 3.8 |
| RenalDisease, % | 3.7 | 2.7 | 2.6 |
| Cancer, % | 8.8 | 8.9 | 7.1 |
| Metastatic Cancer, % | 1.5 | 1.4 | 1.5 |
| SevereLiverDisease, % | 0.1 | 0.1 | 0.1 |
| Glucose, mean (std) | 6.9 (2.6) | 6.6 (2.1) | 6.7 (2.4) |
| Hb, mean (std) | 139.0 (16.5) | 139.7 (16.1) | 139.9 (16.4) |
| Creatinine, mean (std) | 88.1 (42.8) | 81.5 (38.9) | 79.4 (34.0) |
| TnT, mean (std) | 33.4 (219.1) | 29.1 (154.3) | 26.4 (148.7) |
| ECG present, n (%) | 4761 (100.0) | 2379 (100.0) | 2379 (100.0) |
| n, number; AMI, acute myocardial infarction; std, standard deviation; Hb, hemoglobin;  hs-cTnT, high sensitivity cardiac troponin T  * As recorded up to 5 years prior to study event. | | | |

| **Table A7: Comparison of methods - testing set, index visit AMI** | | | | | | | | | |
| --- | --- | --- | --- | --- | --- | --- | --- | --- | --- |
|  | **Rule-out** | | | | **Rule-in** | | | | **Missed AMI** |
|  | **Sensitivity** | **NPV** | **Ruled out** | **(%)** | **Specificity** | **PPV** | **Ruled in** | **(%)** |  |
| ESC 0h | 98.8 | 99.8 | 1123 | 47.2% | 97.1 | 58.9 | 158 | 6.6% | 2 |
| LogReg | 97.6 | 99.6 | 915 | 38.5% | 98.2 | 61.2 | 103 | 4.3% | 4 |
| ANN | 99.4 | 99.9 | 1109 | 46.6% | 98 | 65.1 | 129 | 5.4% | 1 |
| CNN-MB | 99.4 | 99.9 | 1309 | 55.0% | 98.4 | 72 | 125 | 5.3% | 1 |
| CNN-RAW | 99.4 | 99.9 | 1208 | 50.8% | 98.1 | 67.4 | 132 | 5.5% | 1 |
| Performance with respect to rule-out (sensitivity and NPV) and rule-in (Specificity and PPV).  NPV, negative predictive value; PPV, positive predictive value;  ESC 0h, 0h arm of the European Society of Cardiology algorithm;  LogReg, logistic regression; ANN, artificial neural network, CNN-MB, convolutional neural network trained on median beat ECG data;  CNN-RAW, convolutional neural network trained on raw ECG data. | | | | | | | | | |

| **Table A8 Correlation between missingness of values and outcome** | | | |
| --- | --- | --- | --- |
| Missing (%) | 30d AMI or death | Neither AMI nor Death | p-values |
| Hb | 21.1% | 24.0% | 0.15 |
| Glucose | 3.0% | 4.2% | 0.11 |
| Creatinine | 2.0% | 3.1% | 0.07 |
| hs-cTnT | 2.3% | 2.9% | 0.37 |
| ECG | 0.7% | 0.5% | 0.33 |
| hb, Hemoglobin; hs-cTnT, high sensitivity TroponinT | | | |

***References Appendix***

1. Radiometer Medical ApS. ABL800 FLEX Reference Manual. [Internet]. 2008 [cited 2021 Oct 1]. Available from: http://www.healthandcareni.net/stlabs/webhb/poct/documents/poct%20abl800%20man.pdf

2. Medicinsk Service Skåne. B-Hemoglobin (Hb), B-Erytrocyter, Erc-MCV, Erc-MCH, Erc-MCHC, B-EVF, Sysmex XN-10. Rev ed 6. [Internet]. 2021 [cited 2021 Oct 1] .Available from: http://analysportalen-labmedicin.skane.se/pics/Labmedicin/Verksamhetsomr%E5den/Klinisk%20kemi/Analyser/Skane/B-Hemoglobin%20(Hb),%20B-Erytrocyter,%20Erc-MCV,%20Erc-MCH,%20Erc-MCHC,%20B-EVF,%20Sysmex%20XN-10%20samt.pdf

3. Medicinsk Service Skåne. P-Kreatinin på Cobas (NPU04998). Rev ed 13. [Internet]. 2020 [cited 2021 Oct 1] .Available from: <http://analysportalen-labmedicin.skane.se/pics/Labmedicin/Verksamhets>omr%E5den/Klinisk%20kemi/Analyser/Skane/P-Kreatinin%20p%E5%20Cobas%20(NPU04998).pdf

4. Medicinsk Service Skåne. P-Glukos på Cobas (NPU02192). Rev ed 19. [Internet]. 2020 [cited 2021 Oct 1] .Available from: http://analysportalen-labmedicin.skane.se/pics//Labmedicin/Verksamhetsomr%c3%a5den/Klinisk%20kemi/Analyser/Skane/P-Glukos%20p%c3%a5%20Cobas%20(NPU02192).pdf

5. [Bergstra J, Bengio Y. Random Search for Hyper-Parameter Optimization. *J Mach Learn Res*. 2012;13(10):281-305](https://www.zotero.org/google-docs/?broken=LGFnbz)
